# In-depth analysis of secretome and N-glycosecretome of human hepatocellular carcinoma metastatic cell lines shed light on metastasis correlated proteins

**DOI:** 10.18632/oncotarget.8247

**Published:** 2016-03-21

**Authors:** Xianyu Li, Jing Jiang, Xinyuan Zhao, Yan Zhao, Qichen Cao, Qing Zhao, Huanhuan Han, Jifeng Wang, Zixiang Yu, Bo Peng, Wantao Ying, Xiaohong Qian

**Affiliations:** ^1^ National Center for Protein Sciences Beijing, State Key Laboratory of Proteomics, Beijing Proteome Research Center, Beijing Institute of Radiation Medicine, Beijing, China; ^2^ Beijing Key Laboratory of Traditional Chinese Medicine Basic Research on Prevention and Treatment for Major Diseases, Experimental Research Center, China Academy of Chinese Medical Sciences, Beijing, China

**Keywords:** hepatocellular carcinoma, metastasis, secretome, OFFGEL fractionation, zic-HILIC, label-free quantitation, N-glycoproteomics

## Abstract

Cancer cell metastasis is a major cause of cancer fatality. But the underlying molecular mechanisms remain incompletely understood, which results in the lack of efficient diagnosis, therapy and prevention approaches. Here, we report a systematic study on the secretory proteins (secretome) and secretory N-glycoproteins (N-glycosecretome) of four human hepatocellular carcinoma (HCC) cell lines with different metastatic potential, to explore the molecular mechanism of metastasis and supply the clues for effective measurement of diagnosis and therapy. Totally, 6242 unique gene products (GPs) and 1637 unique N-glycosites from 635 GPs were confidently identified. About 4000 GPs on average were quantified in each of the cell lines, 1156 of which show differential expression (p<0.05). Ninety-nine percentage of the significantly altered proteins were secretory proteins and proteins correlated to cell movement were significantly activated with the increasing of metastatic potential of the cell lines. Twenty-three GPs increased both in the secretome and the N-glycosecretome were chosen as candidates and verified by western blot analysis, and 10 of them were chosen for immunohistochemistry (IHC) analysis. The cumulative survival rates of the patients with candidate (FAT1, DKK3) suggested that these proteins might be used as biomarkers for HCC diagnosis. In addition, a comparative analysis with the published core human plasma database (1754 GPs) revealed that there were 182 proteins not presented in the human plasma database but identified by our studies, some of which were selected and verified successfully by western blotting in human plasma.

## INTRODUCTION

Hepatocellular carcinoma (HCC) is a common malignant neoplasm and a major cause of cancer correlated deaths in Asian countries. Furthermore, HCC is the sixth most commonly diagnosed cancer and the third most common cause of death from cancer worldwide [1]. The high mortality rate for HCC is principally caused by uncontrolled tumor invasion and metastasis [2]. Cancer cell metastasis involves intricate, multi-step processes and various cytophysiological changes, including changes in the crosstalk between cells and the components of the extracellular space [3]. Due to its diverse nature and composition, the components in the extracellular space regulating dynamic cell behavior can serve many functions, such as providing support, segregating tissues from one another, and regulating intercellular communication. An understanding of the composition of the extracellular space also helps in comprehending the complex dynamics of tumor invasion and cancer biology [4]. Secretory proteins are a crucial part of the extracellular matrix that create an environment that is favorable to the disorder in many dis­eases [5, 6]. From a clinical perspective, focusing on proteins that are secreted from these cells is very appealing for diagnostic purposes as these proteins may filtrate into the peripheral blood [7]. Over the past several years, the progress in the analysis of the human plasma proteome has provided a tremendous opportunity for discovering clinical biomarkers [8-10]. However, the prospects of blood proteomics and the discovery of new candidates are challenged by the fact that blood is a very complex body fluid, comprising an enormous diversity of proteins and protein isoforms with a large dynamic range of at least 9-10 orders of magnitude [11, 12]. A more straightforward approach would be the analysis of proteins secreted from homogeneous cell populations. Consequently, the conditioned medium of cell lines has been extensively used for the analysis of secreted cancer proteins.

Tumor cell secreted proteins can be specifically profiled without the depletion of high abundance serum proteins by culturing tumor cells in serum-free conditioned medium for a short duration, collecting the conditioned medium and subjecting the media to proteome analysis, called cell secretome analysis [13, 14]. Over the past several years, efforts have focused on the analysis of cell secretome to identify reliable and useful cancer biomarkers. In a representative study, the secretome of a panel of cancer cell lines were generated, with the detection of 4,600 proteins from 23 cell lines of breast cancer [15, 16]. High-quality quantitative analysis of cancer cell secretome has also been accomplished by combining azidohomoalanine labeling and stable isotope labeling with amino acids in prostate cancer cells [17]. Moreover, quantitative proteomics approaches and comparative secretome strategies have also been used for lung cancer cells analysis [18]. However, there are no reports associated with an in-depth and comprehensive analysis on the metastatic secretome of hepatocellular carcinoma cell lines to date.

Protein glycosylation has been directly linked to cancer development[19]. Almost all of the currently used protein biomarkers are secreted glycoproteins, such as carcinoembryonic antigen (CEA), cancer antigen 125 (CA125), prostate specific antigen (PSA) and alpha-fetoprotein (AFP) [20]. Because most of the proteins in the secretory system are glycosylated [21], it is natural to expect that the glycoproteomic analysis of the tumor cell secretome will provide valuable biomarker candidates. Although many studies have been performed for the in-depth profiling of glycoproteins in the plasma, efforts to profile the glycoproteins of secretory proteins from the conditioned medium (CM) remain preliminary [7]. Only limited research has been performed to explore the N-glycosylation changes of the secretome that are derived from hepatocellular carcinoma cells, despite significant biological and clinical interests [22].

Here, we report a comprehensive delineation of the secretome and N-glycosecretome of HCC cell lines that differ only with respect to their metastatic potential (no (Hep3B), low (MHCC97L), and high (MHCC97H, HCCLM3) metastatic potential). The latter three cell lines are derived from the same genetic background [23]. Combining IEF for peptides fractionation and LC-MS/MS for protein identification, a largest secretome dataset with 6242 unique Gene Products (GPs) was confidently identified in three biological replicates. A label-free approach was then used to quantify the differences in the signal intensity of the MS response between the HCC metastatic cell lines. Hierarchical clustering and bioinformatics analysis revealed that 297 unique GPs identified above were differentially expressed and potentially involved in the process associated with cancer development and metastasis. Meanwhile, using a combination of ^18^O labeling, FASP digestion and HILIC enrichment with LC-MS/MS identification, a total of 1,637 unique N-glycosites from 711 unique glycoproteins (mapping to 635 GPs) were confidently identified; 649 N-glycoproteins (604 GPs) were mapped to biological processes (BP) by Gene Ontology (GO) annotations, and 303 GPs focused on the “cell adhesion & migration” cluster. Combined with the increased results from the two hierarchical clusters of the secretome and N-glycosecretome, the 23 overlapping GPs were chosen as candidates for validation with western blot and tissue array immunohistochemistry (IHC). Six proteins were verified with respect to their potential as biomarkers for HCC metastasis. Meanwhile, a comparative analysis was conducted to determine whether the differentially expressed proteins discovered by our study could be found in human plasma.

## RESULTS AND DISCUSSION

### Cell line selection

The human metastatic HCC cell lines and control cell line Hep3B were obtained from the Liver Cancer Institute of Zhongshan Hospital, Fudan University (Shanghai, China). Three stepwise human metastatic HCC cell lines with a similar genetic backgrounds, including two subclones with high (MHCC97H) and low (MHCC97L) metastatic potential from MHCC97 [23, 24] and one even higher metastatic potential cell line (HCCLM3) from MHCC97H, were established using an *in vivo* clonal selection procedure [25]. The pulmonary metastatic rate was 100% in MHCC97H and HCCLM3 vs. 40% in MHCC97L [26]. After orthotopic implantation of HCCLM3 tumor tissue into nude mouse livers for 35 days, widespread loco-regional and distant metastases were observed in the lungs and abdominal walls of 100% of the mice, in the intra-abdominal cavity in 80% of the mice, and in the diaphragm in 70% of the mice [24, 26]. However, the pulmonary metastatic rate of MHCC97L was only 40 % [27]. Immunocytochemical studies demonstrated that three clones were positive for AFP, and the concentration of serum AFP was higher in HCCLM3-inoculated mice than in those inoculated with MHCC97L [28]. The cell lines with different metastatic potentials provide an important model system for the *in vivo* and *in vitro* study of HCC metastasis [29]. Additionally, Hep3B, a p53-null cell line, served as a control due to its nonmetastatic or very low metastatic potential compared with the other three HCC cell lines [30].

### Workflow of the secretome and N-glycosecretome of HCC cell lines

**Figure 1:**
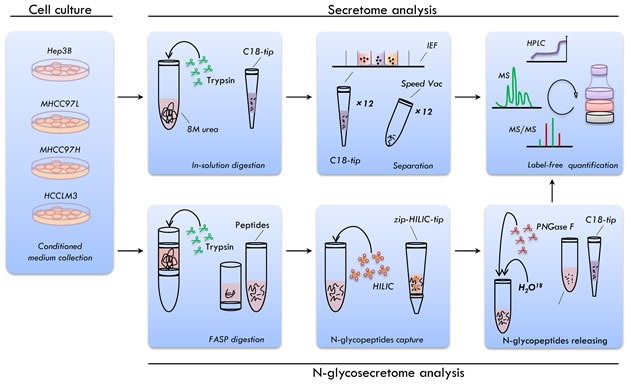
Overview of the experimental workflow The secretory proteins were collected and the secretome and N-glycosecretome were profiled for the four HCC cell lines. Peptide prefractionation was performed using the isoelectric focusing (IEF) electrophoresis. The N-glycosylated peptides were enriched using zwitterionic hydrophilic interaction chromatography (zic-HILIC) methods. The peptide mixture was analyzed with online reversed-phase chromatography and mass spectrometry and label-free approach was used for the quantitative analysis.

Figure 1 is a schematic representation of our experimental approach. An initial experiment revealed that none of the metastatic cell lines suffered from significantly reduced viability after 24 h of culturing in the serum-free medium [31]. Thus, the culture time of 24 h in DMEM CM without serum was identified as the essential conditions for culturing the cells [23]. The CM was centrifuged and precipitated with TCA. Proteins for secretome analysis were tryptic digested in solution, desalted using a C18 pre-column, and subjected to an OFFGEL fractionator. The OFFGEL fractionator assists in pI-based fractionation using immobilized pH gradient strips [32-34]. We collected 12 fractions from the OFFGEL fractionator for each cell line and desalted the fractions using a C18 tip prior to LTQ-Orbitrap analysis. To ensure the reliability of the quantitative profiling results, the samples were prepared and fractions were collected on three occasions (three biological replicates).

N-glycosecretome protein digestion was performed using the filter-aided sample preparation (FASP) method [35, 36]. After digestion, the N-glycopeptides were captured using the HILIC material. Next, the enriched N-glycopeptides were processed by PNGase F in H_2_^18^O, which resulted in a 2.9848-Da increase in peptide mass because of the introduction of an ^18^O atom during the PNGase F cleavage [7, 37, 38]. The deglycosylated peptides were then characterized using a Q Exactive mass spectrometer. The MS data were searched against the RefSeq Human database (20120320). The number of peptide spectral matches (PSMs) and the matched precursor ion area were further separately used for label-free quantification.

### Optimization of cell culturing in the conditioned medium

HCC cells were grown in serum-free medium (CM) to ensure that the environment of HCC cells contained no other exogenous proteins. The most optimal conditions for growth contained that: after culturing in CM for 1 h, the cells were washed four times with DPBS and four times with serum-free DMEM (CM) before culturing for an additional 24 h. Under such conditions, the concentration of the serum could be markedly reduced (Supplemental Figure S1A). The total number of cells alive at the end of harvest was average 1 × 10^7^ and the secretory proteins were recovered for each experiment (Supplemental Figure S1B). Moreover, Nearly 0.1 LDH units average measurement indicated that less than 1% cell death occurred under the experimental conditions (Supplemental Figure S1C), which is a significant improvement compared with the early reported value of ∼ 6-7% [39, 40]. In addition, we added the experiment of 10% TritonX-100, Normal culturing as the control, the results confirmed the description above and shown that far less than 1% cell death occurred under our experimental conditions.

### Identification and quantification of the secretome

**Figure 2:**
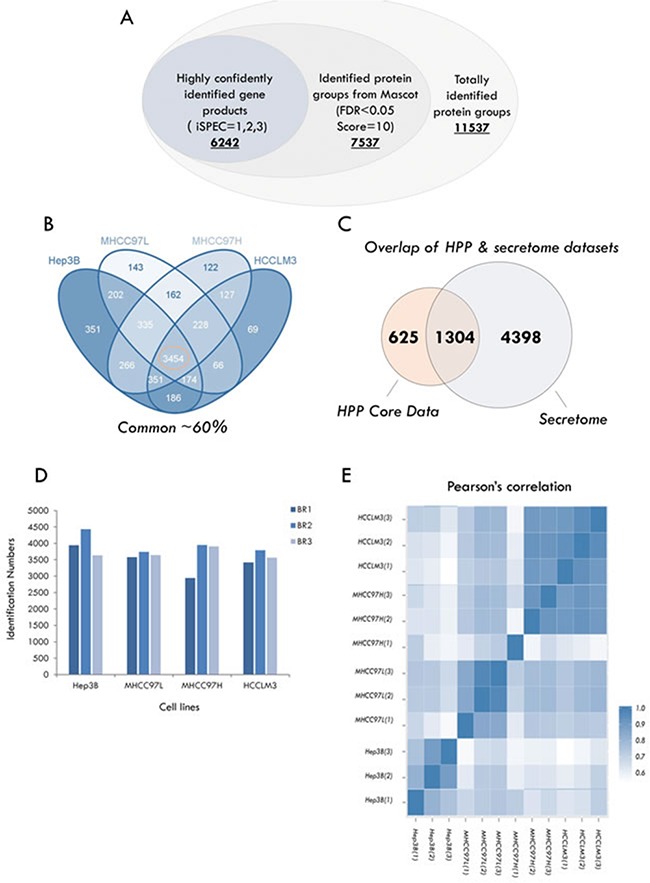
Summary of identification and quantitative analysis of the secretome **A.** The rigorous evaluation of the dataset by applying the parsimony principle filter in peptide grouping.**B.** The number of highly confident genes products (GPs) identified in HCC cell lines. **C.** Overlap of proteins between the different HCC cell lines. **D.** Overlap of proteins identified between the HCC secretome dataset and core human plasma database. **E.** Pearson correlation coefficients between the measurements of four HCC cell lines.

Combining the raw data from 144 MS runs (4 cell lines, 12 fractions for each, 3 biological replicates), a total of 11,537 proteins (q<0.05 from PD software) were identified. Applying stepwise stringent quality control strategies, over 6500 proteins (representing 6242 GPs) were identified (Figure 2A) (Supplemental Table S1), 3454 GPs of which (∼60%) were common among the HCC cell lines (Figure 2B). Matching our secretome dataset to the core human plasma database, which contains 1929 proteins [41], revealed that almost 70% (1304 GPs) of the proteins were covered by the HCC secretome (Figure 2C).

In each of the cell lines, we quantified ∼4000 GPs on average (Figure 2D). Meanwhile, we calculated the Pearson correlation coefficients between the ratios of the individual samples [42]. Quadruplicates of the HCC cell lines exhibited very tight correlations (Figure 2E), indicating the reproducibility and precision of the quantitative strategy. Next, we tested significant protein expression between the four HCC cell lines with three biological replicates using SPSS analysis (One-way ANOVA, significantly different with *p* < 0.05), and the expression of 1156 GPs exhibited significant differences.

### Biological categorization of significantly altered proteins

One major work is to establish a rationalized method to reliably differentiate this subset from intracellular contaminants, especially in *in vitro* conditions. To address this issue, we deployed a three-step analytic procedure based on predictions from classical (Signal P) or non-classical (Secretome P) protein secretion and the presence of transmembrane helices (TMHMM). Then, we categorized all proteins into four categories depending on the scores that each protein received throughout the procedure (Supplemental Figure S2A) [43, 44]. The 1156 proteins were divided into the following groups: classical secretome proteins (Part 4), extracellular membrane proteins (Part 3), non-classical secretome proteins (Part 1) or other proteins (Part 2) (Supplemental Figure S2A&B). For further analysis, we only kept proteins in groups 1, 3 and 4. Interestingly, proteins in group 2 only account for 14% of the differently expressed proteins, which indicates that most of the differently expressed proteins are bona fide secretory proteins (Supplemental Figure S2B). All of the proteins in group 2 belong to the exosome as revealed by comparison with an exosome database [45]. In particular, classical secreted proteins account for ∼ 21% of the differentially expressed proteins, and non-classical secreted proteins and membrane protein account for ∼52% and ∼14%, respectively. Based on the literature evidence [39, 40, 46-48], 99% of the significantly altered proteins were predicted to be secretory proteins.

To obtain a general overview, we analyzed the 1156 significantly differentially expressed proteins using IPA analysis tools for molecular functions and MetaCore tools for cellular localizations enrichment. From the IPA, the top 9 enriched functional categories in the metastatic cell lines are presented in Supplemental Figure S2C. The top enriched categories were correlated to cell movement, which could be involved in cancer cell migration and invasion (*p*<0.001 and z-Score >2). For instance, the categories included the migration of tumor cell lines, the movement of tumor cell lines, the invasion of tumor cell lines and the organization of the cytoskeleton, all of which were significantly activated. The cellular localizations of these proteins are presented in Supplemental Figure S2C. Among the most common cellular localizations, the top three enriched categories were “extracellular region”, “extracellular space” and “membrane-enclosed lumen”, and which further indicated that the dataset is a high-quality secretory protein collection.

### Hierarchical clustering of the significantly altered proteins

**Figure 3:**
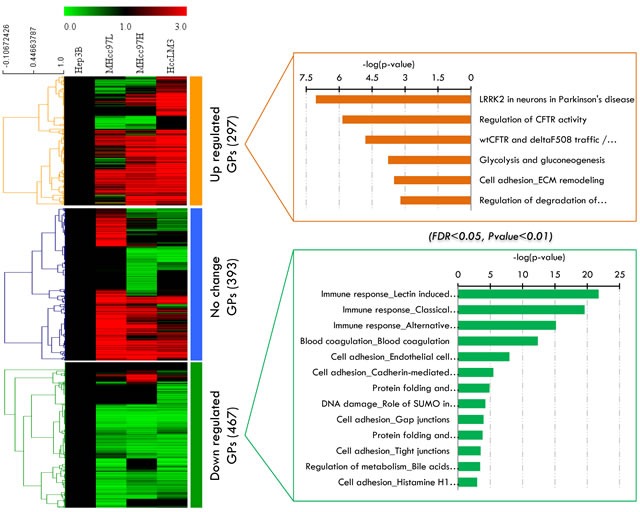
Label-free quantitative analysis of significant altered proteins in the secretome analysis **A.** Three major clusters extracted from these proteins by K-Means clustering. **B.** The enriched biological pathways in cluster 1 and cluster 3 viewed by MetaCore.

A significant difference (*p*<0.05) in the 1156 secretome proteins in at least one of the cell lines was taken as an excellent basis for further statistical analysis. We then performed hierarchical clustering based on Pearson correlation of quantitation (Figure 3A) and the k-means values of the proteins per cell lines. Three main clusters can be observed with increased, decreased or unchanged proteins (Supplemental Table S2). In cluster 1, the expression levels of secretome proteins (297 GPs) continuously increased with the metastasis level of the HCC cell lines. Cluster 2 contains one third of the identified secretome proteins with mixed regulation and without any significant changes in abundance among the four HCC cell lines. Cluster 3, which represents nearly 50% of the dataset (467 GPs) was continuously decreased in metastatic cell lines. To investigate this more systematically, we analyzed the hierarchical clusters (cluster 1 and cluster 3) of the 1156 proteins and their quantification information, which we then used as an input to view enrichment pathways by MetaCore (Figure 3B). The high connectivity of the pathways “immune response” (*p*-value= 2.550E-20 and FDR= 6.376E-18) and “blood coagulation” (*p*-value= 4.356E-13 and FDR= 5.446E-11) were enriched in cluster 3, whereas cluster 1 exhibited significantly more proteins within the “glycolysis” ( *p*-value= 1.701E-04 and FDR= 1.205E-02) and “ECM remodeling and cell adhesion” (*p*-value= 3.156E-04 and 1.957E-02) pathways. The fact that the abundance of latter cluster is lower in the no-metastasis cell lines suggests the positive involvement of these proteins in the release of cells from the surrounding tissue and supports cell metastasis. Meanwhile, the decrease proteins in cluster 3 indicates the reduced expression of such genes in HCC cells with no metastasis ability. From a biomarker perspective, cluster 1 can be used as a positive (increasing) candidate dataset for HCC metastasis development.

### General characteristics of the N-glycosecretome of HCC cell lines

The details of the protein identifications are described in Supplemental Table S3. Two biological replicates were conducted. Compared with other studies using similar enrichment methods [7, 34, 38], higher numbers of N-glycosites and glycoproteins were identified from the HCC cell line secretome here (1,637 unique N-glycosites and 711 unique glycoproteins (mapped to 635 GPs) from the zic-HILIC method) (Supplemental Figure S3A). All N-glycosites we identified from the HCC secretome match the canonical N-!P-[S/T] motif (Supplemental Figure S3B). Approximately 49 % of the glycoproteins were identified with one N-glycosite, nearly 23 % with two or three N-glycosites, and approximately 17 % with four to nine N-glycosites. Very few proteins (11 proteins) were identified with more than ten sites (Supplemental Figure S3C). The protein with the most N-glycosites was low-density lipoprotein receptor-related protein 1 (LRP1), with 28 identified N-glycosites. Insulin-like growth factor 2 receptor was identified with 14 N-glycosites that bind insulin-like growth factor 2 and the cell surface. Other proteins of regulatory interest included heavily N-glycosylated as well; for instance FN1, NRCAM, FAT1 and LAMA5 each have more than ten sites and also belong to a family of extracellular glycoproteins.

The overlap of unique N-glycosites and unique N-glycoproteins between the four HCC cell lines can be observed in Supplemental Figure S3D. Based on the HILIC enrichment methods, an average of 900 unique N-glycosites and 460 unique N-glycoproteins were detected from each cell lines, and the degree of overlap for the N-glycosites and N-glycoproteins was ∼25 % and ∼53 %, respectively. The overlap among the metastatic cell lines for the N-glycosites and N-glycoproteins was ∼53 % and ∼72 %, respectively, which suggests a relatively high degree of similarity between the three HCC metastasis cell lines.

N-glycopeptide quantities were estimated by precursor ions areas, which are presented in Supplemental Table S4. The differentially expressed glycopeptides between the four HCC cell lines are presented in Supplemental Table S5, of which 1,382 N-glycopeptides exhibited quantification values. To avoid the possible interference from noise, the glycopeptides were normalized by the total precursor area of the identified peptides [34, 49].

### Annotation map and profiling of the N-glycosecretome

**Figure 4:**
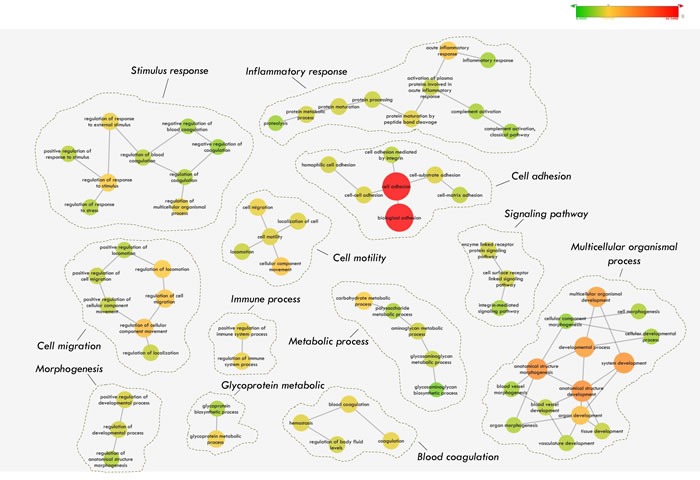
Gene Ontology (GO) annotation of the N-glycosecretome

We wished to investigate whether our proposed N-glycosecretome includes key factors or mediators that may activate or inhibit metastasis during HCC development and progression. Therefore, we performed enrichment map profiling in our N-glycosecretome quantification dataset. In this systemic approach, we used Gene Ontology (GO) annotations for biological processes to map our 649 N-glycoproteins (604 GPs) from 1,382 N-glycopeptides (Figure 4). This analysis resulted in the significant (*p*<0.05) over-representation of 692 GO terms (Supplemental Table S6). Because several annotations are branched together, we visualized the analysis as an enrichment network, which algorithmically clustered GO terms with highly similar content using the enrichment map plug-in in the Cytoscape environment [44, 50-52]. A higher significance level (Benjamini & Hochberg corrected p-value < 1×10^−7^) was selected for our next analysis. A few of the resulting clusters corresponded to “morphogenesis”, “cell motility” “metabolic process” and “inflammation response”, which are considered traditional marks of cancer development and progression. Meanwhile, a rather impressive observation is the overrepresentation of the “cell adhesion”, “cell migration” and “multicellular organismal process”, whose importance in cell metastasis and progression has been extensively reported [51-54] (Benjamini & Hochberg corrected *p*-value =4.62×10^−44^, 3.84×10^−13^, 1.24×10^−13^ & 5.82×10^−13^). Particularly for the above three biological process, well-known hepatocellular carcinoma biomarkers such as alpha fetoprotein (AFP) and Dickkopf-related protein 1 (DKK1) [55] were also identified in the cell adhesion cluster. Finally, several other clusters corresponded to specific biological process, such as “signaling pathway”, “blood coagulation”, “immune response”, “stimulus response” and “glycoprotein metabolism”, the implications of which is also significant in cancer development and progression (Figure 4).

To address the large-scale N-glycosecretome data, an underscored reproducible and biologically relevant criteria for candidate selection were used [56, 57]. As a proof-of-concept, here, we will exclusively focus on the “cell adhesion” clusters of the enrichment map because of its high significance and confidence (Benjamini & Hochberg corrected *p-*value =4.62×10-44). Due to the variety of glycoproteins involved in the regulation of cell-to-cell or cell-to-matrix adhesion, we selected the regulatory glycoproteins from the cluster that could be potentially associated with HCC metastasis and which could potentially serve as markers of HCC metastasis. The other biological processes, such as “multicellular organismal process”, where well-known hepatocellular carcinoma biomarkers AFP and DKK1 was also presented, were used to as the positive controls for our strategy, and the results indicate that the N-glycosylated secretome of the metastatic cell lines provides a potential source of disease markers [34, 55]. Thus, by focusing on the “cell adhesion & migration” and “multicellular organismal process” cluster, we narrowed the 649 N-glycoproteins (604 GPs) of the enrichment map down to 303 N-glycosecretome GPs with 768 N-glycosites for subsequent analysis.

### The secretome versus the N-glycosecretome

We next compared our data set of N-glycosites and proteins to the in-depth secretome of the same HCC metastatic cell lines. Of the 6242 GPs in the secretome, 552 were also found in the 635 N-glycosecretome (Supplemental Figure S4A). Strikingly, 83 GPs were exclusively identified by their N-glycopeptides, attesting to the enrichment capacity of the workflow. In eukaryotes, N-linked glycosylation occurs on secreted or membrane bound proteins, which are often of low abundance, making them more difficult to detect in highly complex samples such as total cellular lysate[42]. In our experiment, compared with those where the corresponding protein was identified in the in-depth HCC secretome, N-glycosecretome examination can reduce complexes of proteins, which was supported by the fact that the de-glycosylated peptides of proteins were only identified in the N-glycosecretome experiment (83 GPs). From PANTHER classification chart analysis [58], the 83GPs were also localized in extracellular regions, the extracellular matrix and the membrane (47.8%, 30.4% and 4.3%, respectively), and 82.5% represented a new contribution for the secretome dataset through our secretome enrichment strategy (Supplemental Figure S4B).

To obtain the validation candidates, we used the same method of hierarchical clustering to filter the 768 N-glycosites of the former secretome, 280 N-glycosites of which were commonly expressed in these HCC cell lines. We then performed hierarchical clustering based on Pearson correlation of ratio of quantitation and the k-means values of the 280 N-glycosites per cell lines (Supplemental Figure S4C). Two main clusters can be observed and included increased and decreased proteins. In cluster 1, the expression of N-glycosites continuously increased with the metastatic level of the HCC cell lines, which includes 112 GPs with 180 N-glycosites.

Combining the increased results from the two hierarchical clusters of the secretome and N-glycosecretome, 23GPs were chosen as validation candidates.

### Selection and validation of the candidates for HCC metastasis by western blotting

**Figure 5:**
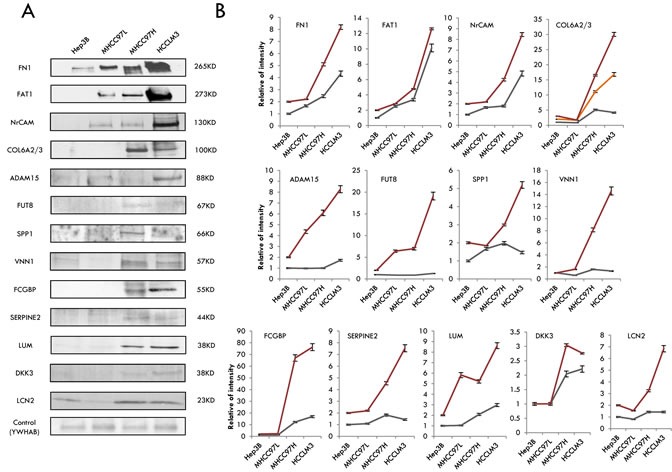
Selection and validation of the candidates for HCC metastasis by western blotting **A.** The results of western blotting of the 13 differentially displayed secretome and N-glycosecretome GPs. **B.** The trends of the protein abundance, either from western blotting or from the label-free analysis. The gray lines stand for the WB results and the red or yellow lines represent the MS data.

To select candidates for HCC metastasis from the 23 differentially expressed secretome and N-glycosecretome GPs, we first applied quantitation data and biological information analysis to confirm their differential expression at the protein level in the four HCC cell lines. All of the 23 proteins passed our subcellular localization criteria as being truly secretome proteins. Thus, we narrowed our list down to eighteen proteins, including the following: Fibronectin 1 (FN1), Protocadherin Fat 1 precursor (FAT1), Neuronal cell adhesion molecule (NrCAM), Collagen alpha-2/3 (COL6A2/3), Disintegrin and metalloproteinase domain-containing protein 15 (ADAM15), Pantetheinase (VNN1), Alpha-(1,6)-fucosyltransferase (FUT8), IgGFc-binding protein (FCGBP), secreted phosphoprotein 1 (SPP1), Glia-derived nexin (SERPINE2), Lumican (LUM), Dickkopf-related protein 3 (DKK3), and Lipocalin-2 (LCN2). Among these GPs, protein Osteopontin encoded by the SPP1 gene was previously reported to indicate a relationship with HCC metastasis [59]. Further, we used western blotting to explore whether the metastatic ability of HCC cells was correlated with the expression of the above proteins. Quantity One software was used to evaluate the western blotting results, and protein 14-3-3 protein beta/alpha (YWHAB) was selected as a loading control because it is expressed equally in the cell lines [18] and because its ratio was also close to 1 based on the mass spectrometry (MS) data. As a result, among the eighteen proteins, thirteen were observed to be highly expressed in HCCLM3 and MHCC97H and expressed at a median level in MHCC97L and a low level in Hep3B cells (Figure 5A), which coincided with their invasiveness and metastasis capability. However, this correlation was not observed for the other proteins (five proteins and the data not show). In addition, many studies have suggested that correct candidates should participate in relevant interactions and be migration/invasion-related proteins. To further validate the up-regulation of proteins at the protein level, we examined the protein abundance via western blotting, compared the western blot results with their MS label-free expression individually (Figure 5B). As expected, the western blotting results of all these proteins were gradually increased among the four HCC cell lines, consistent with the label-free results by MS, lending significant confidence for the next validation step. Ten of the proteins with the best corresponding expression trend between WB and label-free MS were chosen for immunohistochemistry validation.

### Validation of the candidates by immunohistochemistry in tissue microarrays

**Figure 6:**
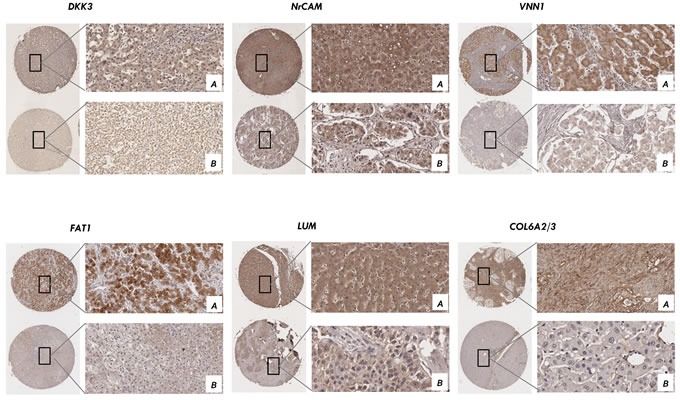
Validation of the candidates by immunohistochemistry in tissue microarray Positive immunoreactivity for six secretome proteins was observed primarily in the cytoplasm and one in extracellular matrix using tissue array from 75 cases of liver cancer patients. Representative IHC from the positive samples (magnification ×200) **A.** and the negative samples (magnification ×200) **B.** are shown.

To explore whether these candidates could be an important factor in determining clinical outcomes of HCC patients, we examined the expression level of these proteins in 150 samples of a tissue microarray from HCC patients with different pTNM stage. Positive immunoreactivity for nine secretome proteins was observed primarily in the cytoplasm and for one protein in the extracellular matrix (Figure 6). The expression of nine proteins (FAT1, NrCAM, ADAM15, VNN1, GALNT5, FCGBP, SERPINE2, LUM, DKK3) was scored by two experienced pathologists from the Chinese Academy of Medical Sciences Cancer Hospital in HCC tissues, but the expression of collagen alpha-2/3 (COL6A2/3) was not scored in the extracellular matrix. Of 75 HCC samples, 90% were highly positive for these proteins, whereas the majority of non-metastatic HCC samples were negative (*p*<0.05 [*χ*^2^ test]). Segregation of these patients into the candidate-positive and candidate-negative groups did not reveal significant correlations with clinicopathological parameters of sex, age, maximal tumor size, liver cirrhosis or venous invasion. However, pTNM stage and tumor differentiation were significantly corrected with NrCAM (Supplemental Tab. 7; *p*<0.05), VNN1 (Supplemental Tab. 8; *p*<0.05), and LUM (Supplemental Tab. 9; *p*<0.05), and pTNM stage was significantly correlated with ADAM15 (Supplemental Tab. 10; *p*<0.05), FAT1 (Supplemental Tab. 11; *p*<0.05) and DKK3 (Tab. 1; *p*<0.05), which were considered to reflect the malignant phenotype of HCC.

**Table 1:** The relationship between the clinical pathological features of HCC and DKK3

Clinicopathological variables	Tumor DKK3 Expression		*P*-Value*
	Positive	Negative	
**Sex**			
Male	22	42	0.185
Female	1	10	
**Age**			
≤50	9	18	0.853
>50	15	33	
**Maximal tumor size**			
≤5cm	19	29	0.060
>5cm	5	22	
**Liver cirrhosis**			
Absent	12	33	0.534
Present	10	20	
**Venous invasion**			
Absent	19	47	0.217
Present	5	4	
**Tumor differentiation**			
I-II	3	6	0.645
II	12	31	
II-III	9	14	
pTNM Stage**			
I	13	10	0.007
II	7	19	
IIIA	1	10	
IIIB	2	6	
IIIC-IV	1	6	

Candidate expression was scored independently by two pathologists. Intensity of staining was scored as 0 (negative), 1 (weak), or 2 (strong), and the extent of staining was based on the percentage of positive tumor cells: 0 (negative), 1 (1%to 25%), 2 (26% to 50%), 3 (51% to 75%), and 4 (76% to 100%). The final score of each sample was assessed by summarizing the result of intensity and extent of staining. Therefore, each case was finally considered negative if the final score was 0 to 1 (±) or 2 to 3 (±) and positive if the final score was 4 to 5 (±) or 6 to7 (±), respectively.

HCC with microscopic portal vein tumor thrombosis or macroscopic portal veinthrombosis indicates tumor venous invasion.

Grading of differentiation status was performed according to the method of Edmondson and Steiner. The tumors were classified into two groups: well differentiated (grades I and II) and poorly differentiated (grades III and IV), by pathological examination

* Statistical analyses were conducted with Fisher's exact test for all the parameters. *P* values less than 0.05 were considered statistically significant.

** The pTNM classification for HCC was based on The American Joint Committee on Cancer/International Union Against Cancer staging system (7th edition, 2002).

**Figure 7:**
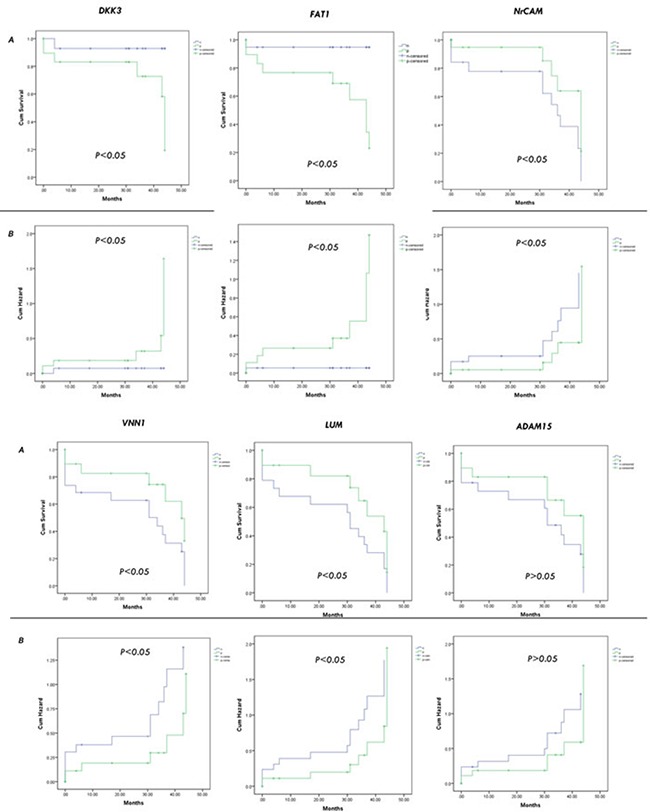
Validation of the candidates by cumulative survival rates analysis The 10-month, 20-month, and 30-month cumulative survival **A.** and hazard **B.** rates of patients with the candidates (FAT1, DKK3, NrCAM, LUM, VNN1, ADAM15).

Furthermore, we investigated the correlation of these candidates with the prognostic data of nineteen patients. Patients with candidate-positive HCC exhibited significantly worse prognosis than those with candidate-negative HCC. The 10-month, 20-month, and 30-month cumulative survival rates of patients with candidate-positive (FAT1, DKK3) HCC were significantly lower than those of patients with candidate-negative HCC, whereas the survival rates of candidate-positive HCC (NrCAM, LUM, VNN1) were significantly higher than those of patients with candidates-negative HCC (Figure 7A; *p*<0.05). The results indicate that FAT1 or DKK3 may be used as novel prognostic biomarkers of HCC.

### Complementary to the human plasma proteome database

A comparative analysis with the published core human plasma database (1754 GPs) [41] revealed that there were 182 proteins not presented in the human plasma database but identified by our studies. Several proteins were verified successfully by western blotting in human plasma, including FAT1, DKK3, ADAM15, LUM and VNN1 (Supplemental Figure S5).

## CONCLUSION

Metastasis remains one of the major challenges for HCC patients undergoing resection. Previous work at the Liver Cancer Institute of Zhongshan Hospital [59] has demonstrated that genes favoring metastasis progression are initiated in the primary tumor. To explore proteins correlated to HCC metastasis, we performed label-free quantitative profiling of the secretomes of HCC cell lines by mass spectrometry. By applying IEF separation and zic-HILIC for glycopeptide enrichment, followed by high mass accuracy LC-MS/MS analysis, secreted peptides and specific N-glycosites were identified with high confidence [18, 37, 38]. The expression of the glycoproteins in the secretory system reflected the intracellular regulation of the N-glycosite synthesis, transport and secretory pathway, which could be illustrated through IPA and MetaCore analysis [60]. Among these biological functions, the most significant class was cell adhesion, which is correlated with cancer cell migration and invasion. Fibronectin (FN1) and extracellular matrix protein 1 (ECM1) are two members of this class that have been previously shown to correlate with the metastasis of other cancer cells [61].

To summarize, our study provides clues for the involvement of a list of proteins in the metastasis processes, and the differentially changed proteins may result in the discovery of novel candidates for the surveillance of tumor metastasis. Further studies must be performed to determine whether these proteins could be used for therapeutic intervention in human hepatocellular carcinoma metastasis.

## MATERIALS AND METHODS

### Cell culture and sample preparation

Approximately 1×10^7^ cells were cultured at 37°C in 5 % CO_2_ in DMEM (Hyclone, USA) supplemented with 10 % fetal bovine serum until reaching 60 - 70 % confluence. Cells were washed stringently and gently four times with Dulbecco's phosphate buffered saline with calcium and magnesium (DPBS) and four times with serum-free DMEM (Conditioned Medium, CM). Cells were then incubated in the CM at 37°C for 24 h. The condition medium was collected and centrifuged at 2,500 ×g for 10 min (4°C) to remove the detached cells and large debris. The resulting supernatant was collected and centrifuged for 1 h at 100,000 ×g (4°C) to remove smaller debris and vesicles. Lactate dehydrogenase (LDH) detection revealed no significant effect on the death of HCC cells using the CM cultured method. Formic acid (FA, final concentration of 0.1 %) was immediately added to the final supernatant, which was then stored at −80°C. The addition of FA lowered the pH (pH < 4) of the culture supernatants, thus reducing the activity of many proteases [22, 62].

The proteins in the culture supernatants were extracted by trichloroacetic acid (TCA) precipitation. Thirty milliliters of culture supernatants were used for each precipitation. All of the treatments were performed at 4°C. TCA was added to the CM solution to a final concentration of 12 % [63]. After mixing, the proteins were precipitated for 4 h at 4°C, followed by centrifugation of the solution (10,000 ×g, 10 min, 4°C). The protein pellet was carefully washed twice with 2 mL tetrahydrofuran (THF) (pre-cooled on ice), and each wash was followed by centrifugation (15,000 ×g, 10 min, 4°C). The final pellet was re-dissolved in 0.4 mL lysis buffer (8M Urea). The concentrations of the extracted proteins were measured by a NanoDrop spectrophotometer (Thermo, USA) at 280 nm with an extinction coefficient of 1.1 absorbance units [35].

### In-solution digestion for secretome protein

Supernatants were collected from cell lysate after centrifugation at 15,000 × g for 5 min at 4°C. The in-solution tryptic digestion was performed using a standard protocol. The lysate were denatured using 8 M urea, reduced with 10 mM DTT and alkylated with 40 mM IAA. Excess IAA was quenched by adding 30 mM of DTT. The urea concentration in the sample solution was reduced to 1 M by diluting the samples with 50 mM NH_4_HCO_3_, and the proteins were digested with trypsin (Promega, USA) overnight. The protein to enzyme ratio was 100:1, and protein digestion was stopped by adding formic acid at a final concentration of 0.1%.

### OFFGEL fractionation

Tryptic peptide samples were desalted using a C18 cartridge (Waters, USA) and fractionated by means of a 3100 OFFGEL fractionator (Agilent Technologies, USA) [34]. Peptides were fractionated according to the manufacturer's protocol using Immobilin^TM^ DryStrip, pH 3-10, 13 cm (GE Healthcare). Twelve fractions were collected from the fractionator. The fractions were desalted using a C18 tip, dried, reconstituted in 0.1% FA, and subjected to MS analysis.

### FASP digestion of N-glycoproteins

Detergent was removed from the lysate, and the proteins were digested with trypsin using the FASP protocol [35], which entails the ultrafiltration of spin units with a nominal molecular weight cut-off of 30,000 daltons. Briefly, 200 μL of 8 M urea in 0.1 M Tris/HCl, pH 8.5 (UA buffer) was added to YM-30 Microcon filter units (Millipore, USA) containing the CM protein concentrates, and the units were centrifuged at 14,000 ×g at 20°C for 15 min. This step was repeated twice. Next, 50 μL 0.05 M iodoacetamide in 8 M urea was added to the filters, and the samples were incubated in the dark for 20 min. The filters were washed twice with 100 μL 8 M UA buffer followed by three washes with 100 μL 50 mM NH_4_HCO_3_. Finally, trypsin (Promega, USA) was added in 100 μL 50 mM NH_4_HCO_3_ to each filter. The protein to enzyme ratio was 100:1. The samples were incubated overnight at 37°C, and the digested peptides were collected by centrifugation at 14,000 ×g at 20°C for 15 min.

### N-glycopeptide enrichment by HILIC

The digested peptides were enriched with zic-HILIC media following the procedure reported by Calvano [64] with slight modifications. Briefly, first, the C8 disk was inserted into a 200-μL tip. Approximately 100 μg of in-solution digested samples was dissolved in 80 % ACN/0.5 % FA and incubated overnight at room temperature with 2 mg zic-HILIC media (Merck; particle size 10 μm) that was pre-washed twice with 1 mL coupling buffer (80 % ACN, 0.5 % FA). Subsequently, zic-HILIC media was loaded into the 200-μL tip that was pre-filled with a C8 disk. The zic-HILIC tip was washed 5 times with 100 μL 80 % ACN/1 % FA/19 % H_2_O, and the bound peptides were eluted 3 times with 80 μL elution buffer (99 % H_2_O, 1 % FA). The eluate was dried, de-glycosylated with 3 μL PNGase F (500 units/μL, New England Biolabs) in 50 mM NH_4_HCO_3_ (dissolved in ^18^O water) at 37°C overnight and dried down to a final volume of 20 μL using a SpeedVac concentrator before analysis on a Q Exactive mass spectrometer (Thermo Fisher Scientific, USA).

MS analysis for peptides and N-glycopeptides identification

LC MS/MS analysis of the secretome peptides was conducted on an LTQ-Orbitrap Velos mass spectrometer (Thermo Fisher Scientific, USA) equipped with an EASY-nano LC system and a nanoelectrospray ion source (Thermo Fisher Scientific, USA). The sample was injected (5 μl) at a flow rate of 300 nl/min into a self-packed precolumn (20 mm, 5 μm, 100°A). Chromatographic separation was performed on a self-packed reversed phase C18 nanocolumn using 0.1% formic acid in water as mobile phase A and 0.1% formic acid in 80% acetonitrile as mobile phase B with a flow rate of 300 nl/min. The full-scan mass range was set from m/z 300-1400 with a resolution of 60,000. The twenty most intense ions were sequentially isolated for MS2 by LTQ. The electrospray voltage was maintained at 1.9 kv, and the capillary temperature was set at 320°C.

A nanoflow HPLC instrument (EASY-nLC 1000 system, Thermo Fisher Scientific, USA) was coupled on-line to a Q-Exactive mass spectrometer with a nanoelectrospray ion source (Thermo Fisher Scientific, USA) for the N-glycopeptides analysis[7]. Chromatography columns were packed in-house with Ultimate XB-C18 3-μm resin (Welch Materials, USA). The peptide mixtures were loaded onto the C_18_-reversed phase column (10 cm length, 75 μm inner diameter, 360 μm outer-diameter ×10 cm, 3 μm C18 resin). A linear gradients from 3% mobile phase B (99.5 % acetonitrile and 0.5 % FA) to 30% mobile phase B with a flow rate of 300 nl/min in total 78min. The electrospray voltage was 2.0 kV. Peptides were analyzed by MS/MS acquisition with a dynamic exclusion duration of 18 s. In MS1, the resolution was 70,000, the AGC target was 3e^6^, and the maximum injection time was 20 ms. In MS2, the resolution was 17,500, the AGC target was 1e^6^, and the maximum injection time was 60 ms. The scan range was 300 - 1400 m/z, and the 20 most intensive precursor ions were selected for MS/MS analysis.

### Protein identification

The raw data were processed using the Proteome Discoverer 1.3 proteomics platform. The fragmentation spectra were searched against the RefSeq Human database (20120320) using the Mascot search engine (v 2.2.06) with the precursor and fragment mass tolerances set to 10 ppm and 20 mmu for QE data and 20ppm and 0.5 Da for LTQ-Orbitrap Velos data, respectively. Two missed cleavage sites were allowed, and the minimum peptide length was 7 amino acids. The variable modifications included oxidation (M) and acetylation (protein N-terminal), and the fixed modification was carbamidomethyl (C).

Additionally, the deamination of asparagine together with the incorporation of an ^18^O atom was set as a variable modification for the assignment of N-glycosites. Peptide ions were filtered using the cut-off scores of Percolator based on *p*-values < 0.01. The false discovery rate (FDR) was set to 1 % for peptide identifications.

### Label-free quantitative analysis

The label-free quantitative analysis of secretome peptides was performed using the FileMaker in-house software based on Proteome Discoverer 1.3 and Mascot. The MS raw data files were processed by Proteome Discoverer to generate the peak lists using default parameters. The peak lists were analyzed by Mascot (v 2.2.06), and the results were exported in csv format. The csv results were transferred into an in-house-built FileMaker-based relational database where protein identification numbers (protein GIs) were converted to the GeneID identifiers according to the NCBI “gene to accession” table. The quantities of identified proteins were estimated by spectral counts method, and data filtering and deconvolution were performed as described [65]. Briefly, the sum of the number of peptides spectral matches (PSMs) of each protein was normalized by its theoretical number of all possible full tryptic peptides. Proteins or gene products (GPs) that are shared by non-unique peptides were not chosen. The normalized PSM was named SAF1 (spectral abundance factor), which was displayed in 10 units for better visual comprehension. The relative quantity of each protein or gene products (GP) was calculated as a fraction of total SAF1, and named NSAF5 (normalized SAF1) (displayed in 10^5^ units). For biological reliability, we performed three biological replicates (totally 144 MS run). In addition, we analyzed the dataset from LTQ Orbitrap Velos using in-house software by applying stepwise stringent quality control strategies (Mascot ion score > 30, peptide FDR < 1%, Mascot ion score > 40, peptide FDR < 5%, applying the parsimony principle filter in peptide grouping).

For N-glycopeptide label-free quantification, precursor ions areas were extracted using Proteome Discoverer 1.3 with 10 ppm mass precision (the experimental m/z and retention times were recorded for precursor area quantification). Ratios for each peptide were normalized by the total identified peptides, and the ratios were used for N-glycopeptide level quantification. Two biological replicates were performed for each cell line.

### Western blotting analysis

The secretory proteins of metastatic HCC cell lines were prepared from serum-free condition medium (CM) and resolved on 10 % SDS-PAGE gels and were subsequently transferred onto PVDF membranes (Millipore, USA). After incubation in blocking buffer (0.5 % Tween-20 in TBS, 5 % BSA) for 1 h at room temperature, membranes were blotted using antibodies against the selected proteins for 1 h at room temperature. Membranes were then washed with TBST (TBS with 0.5% Tween-20) and incubated in 1:2,000 diluted AP-conjugated IgG for 1 h at room temperature. After washing three times with TBST, the bands on the membrane were visualized using a BCIP/NBT detection system [23].

### LDH measurements

LDH is an intracellular enzyme and is an indicator of cell death in the CM. LDH was measured using an enzymatic assay based on lactate to pyruvate conversion and parallel production of NADH from NAD [39, 40]. The LDH experiment process according to the cytotoxicity LDH Assay Kit-WST@ manufacturer' s instructions (DOJINDO, #CK12, Japan), and three controls (DMEM background control, Normal culturing control and 10% TritonX-100 (Sigma) conditions control) experiments were added. 1×105 cells for each well of 96- well plates were culturing in 37°C, 5% CO2 and twenty-four hours, except the well for 10% tritonX-100 process, which was cultured for five hours. Because of the A490nm OD of the cells in tritonX-100 overflowed the range of instrument measurement, five times dilution were used to detection in the end. The production of NADH was measured by spectrophotometry at 490 nm using an automated method (Biotek system, USA).

### TMAs and immunohistochemical staining

Tissue microarrays (TMAs) was constructed as described previously [66, 67]. TMA slides were constructed (Shanghai Biochip, China) using samples from the cohort of 75 HCC patients. A total of 150 cases were used to examine the expression and prognosis of these candidate genes in HCC. Immunohistochemical (IHC) staining was performed as described previously [68]. Briefly, the sections were incubated with mouse anti-human monoclonal antibody at 4°C overnight, followed by staining with an HRP-conjugated secondary antibody. The expression was revealed by the addition of 3, 3-diaminobenzidine (DAB) buffer. PBS was used in place of the primary antibody in all negative controls. The high or low expression of antibody of the proteins of interest in HCC tissues were scored [69] semi-quantitatively and were interpreted according to the staining intensity and the percentage of positive cells by two experienced pathologists from the Chinese Academy of Medical Sciences Cancer Hospital.

### Bioinformatics and statistical analysis

SignalP 4.0 software was used to predict classical amino-terminal secretion signal peptides. Non-classically secreted proteins were predicted using SecretomeP 2.0. Protein THMs were predicted using TMHMM 2.0. All of this software is publicly available from the Centre for Biological Sequence Analysis at the Technical University of Denmark (http://www.cbs.dtu.dk/services/). The following cutoffs were considered a successful pass: 0.5 for SecretomeP (>0.5 or ≤ 0.5) and a 0 score for TMHMM (=0 or ≠0). An automation tool was developed in-house in Perl and can be run on a Windows Perl environment, facilitating the mapping of a list of protein identities to sequences, running the three prediction software programs mentioned above and creating a report file with consolidated predictions [44].

To evaluate the trend of protein abundance changes, hierarchical clustering was performed and a distance tree was generated using Multi Experiment Viewer (MeV) [70]. To explore the biological functions, subcellular localization, pathways and networks of the secretome proteins involved, Gene Ontology (GO) annotation [71], DAVID Bioinformatics Resources [60], Ingenuity Pathways Analysis (IPA, Ingenuity Systems, Mountain View) and MetaCore pathway analysis software were employed, respectively. Subsequently, Cytoscape v.3.0.2 was used to visualize gene-disease interaction networks and cluster function networks [72].

To visualize the significantly changed secretome proteins, Statistical Product and Service Solutions (SPSS 19.0) software was used. For comparisons, one-way analysis of variance (ANOVA) was performed as appropriate. The association between candidate proteins expression and the clinicopathological features of the HCC patients was evaluated by the χ2 test. The cumulative hazard and survival probability were evaluated using the Kaplan-Meier method, and differences were assessed using the log-rank test [73]. All *p-*values reported are two-sided, and *p* < 0.05 was considered statistically significant.

## SUPPLEMENTARY MATERIAL FIGURES AND TABLES
















