# Supplementary material for: In-depth analysis of secretome and N-glycosecretome of human hepatocellular carcinoma metastatic cell lines shed light on metastasis correlated proteins
Source: Oncotarget. 2016 Mar 21;7(16):22031–49. doi: 10.18632/oncotarget.8247 (PMC5008342; doi:10.18632/oncotarget.8247)
Supplement: Supplementary file 1 [file oncotarget-07-22031-s001.pdf]

In-depth analysis of secretome and N-glycosecretome of human hepatocellular carcinoma metastatic cell lines shed light on metastasis correlated proteins

Supplementary Material

Supplemental Table S1

The details of the protein identifications from from LTQ Orbitrap Velos using in-house software by applying stepwise stringent quality control strategies and identified 6242 GPs.

Supplemental Table S2

Three major clusters extracted from these significant altered proteins in the secretome analysis by clustering

Supplemental Table S3

The details of the N-glycoproteins identifications and a total of 1,637 unique N-glycosites and 711 unique glycoproteins (mapped to 635 GPs) were recognized from the zic-HILIC method.

Supplemental Table S4

The details of the N-glycopeptides identifications from the N-glycosecretome, including the peak area information used for label-free quantitative analysis.

Supplemental Table S5

A total of 1,382 N-glycopeptides exhibited quantification values from N-glycosecretome.

Supplemental Table S6

This GO map analysis resulted in the significant ($p<0.05$) over-representation of 692 GO terms.

Supplemental Table 7-11

The relationship between the clinical pathological features of HCC and the five validated candidate proteins.

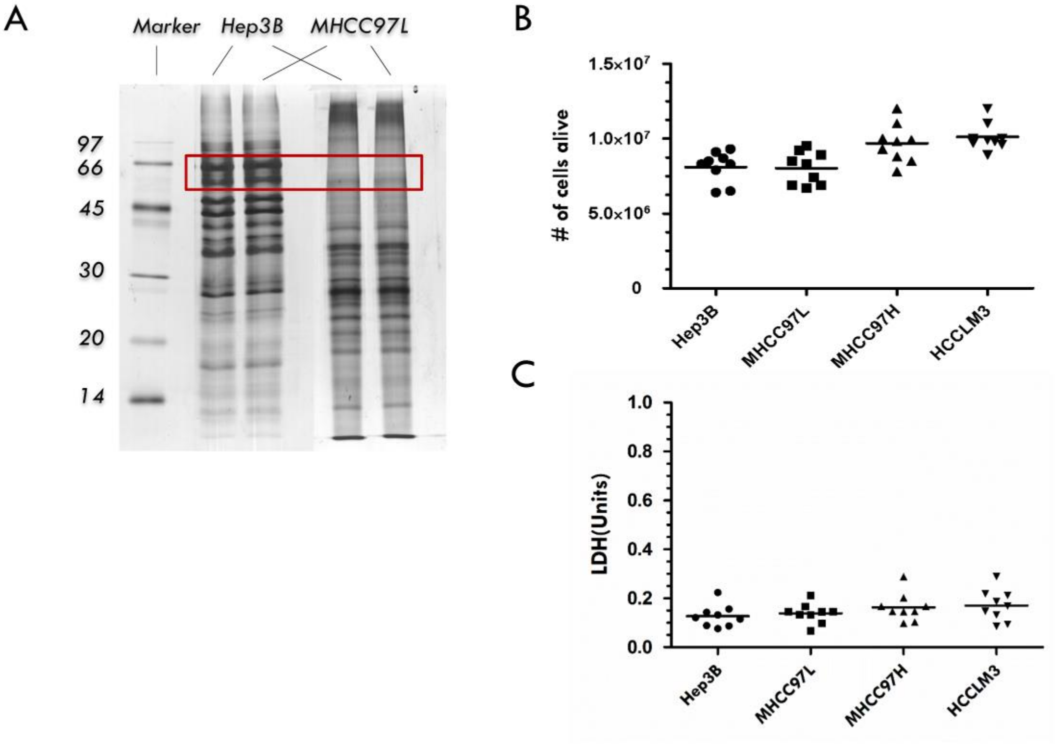

Supplemental Fig. S1

Evaluation of the quality of the collected secretory proteins and the conditioned medium (CM). A) The culturing condition of CM was optimized and the contamination of serum was markedly reduced. B) Approximately 1×10^7 cells were alive after CM culturing and each experiment was repeated over five times. C) LDH level was used to monitor cell death in the CM, which indicated that lower than 1% cell death occurred in the experimental condition. Each experiment was repeated over five times.

B

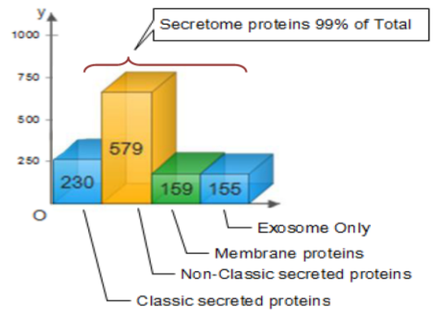
y
1000
750
500
250
O
Secretome proteins 99% of Total
230
579
159
155
Exosome Only
Membrane proteins
Non-Classic secreted proteins
Classic secreted proteins

A

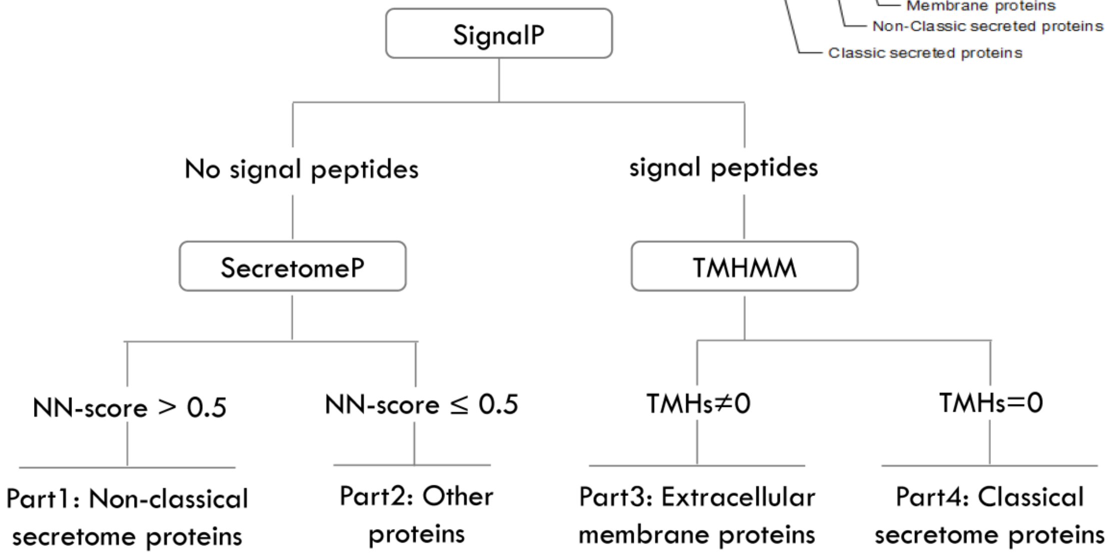
SignalP
No signal peptides
signal peptides
SecretomeP
TMHMM
NN-score > 0.5
NN-score ≤ 0.5
TMHs≠0
TMHs=0
Part1: Non-classical secretome proteins
Part2: Other proteins
Part3: Extracellular membrane proteins
Part4: Classical secretome proteins

Functions annotation from IPA

Functions Annotation	p-Value	Predicted Activation State	Activation z-score
Migration of tumor cell lines	1.54E-04	Increased	2.732
Cell movement of tumor cell lines	1.63E-04	Increased	3.015
Invasion of tumor cell lines	2.90E-04	Increased	2.953
Metabolism of protein	9.77E-04		0.928
Degradation of protein	3.34E-03		1.4
Degradation of protein	3.34E-03		1.4
Organization of cytoskeleton	9.71E-03	Increased	2
Cell viability	3.04E-02		1.299
Cell viability of tumor cell lines	3.09E-02		1.299

Location for identification

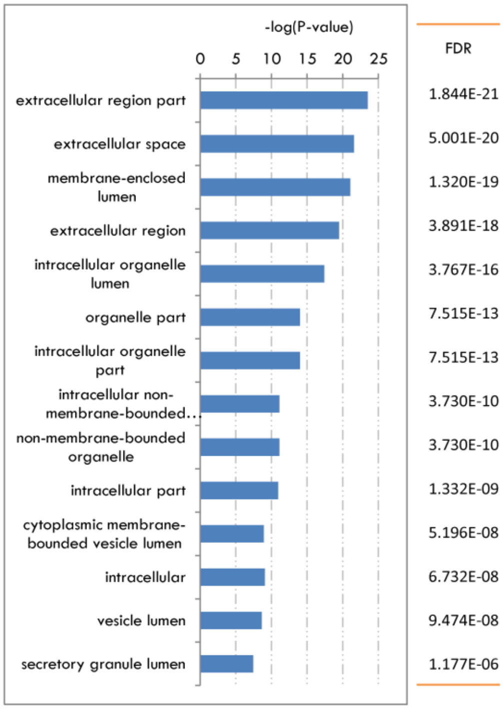

Supplemental Fig. S2

Biological categorization of significant altered proteins. A) A three-step analysis procedure based on predictions from classical (SignalP) or non-classical (SecretomeP) protein secretion and presence of transmembrane helices (TMHMM) was deployed for next step analysis. B) Cellular component annotation of the 1156 significantly expressed proteins. C) Molecular functions and cellular locations of the 1156 differentially expressed proteins.

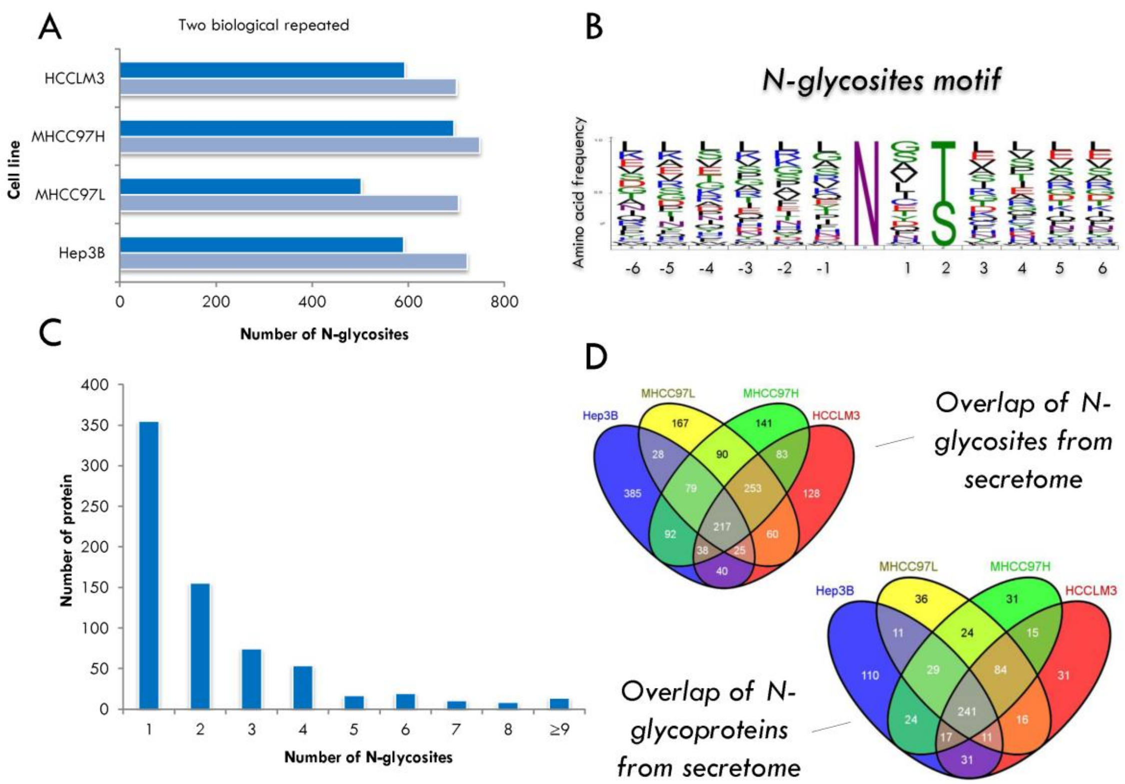

Supplemental Fig. S3

General characteristics of the N-glycosecretome of HCC cell lines. A) A total of 1,637 unique N-glycosites and 711 unique glycoproteins (mapped to 635 GPs) were recognized. B) All N-glycosites we identified from the HCC secretome match the canonical N-!P-[S/T] motif. C) Number of N-glycosites identified per protein. D) Overlap of the N-glycosites and proteins between the different cell lines.

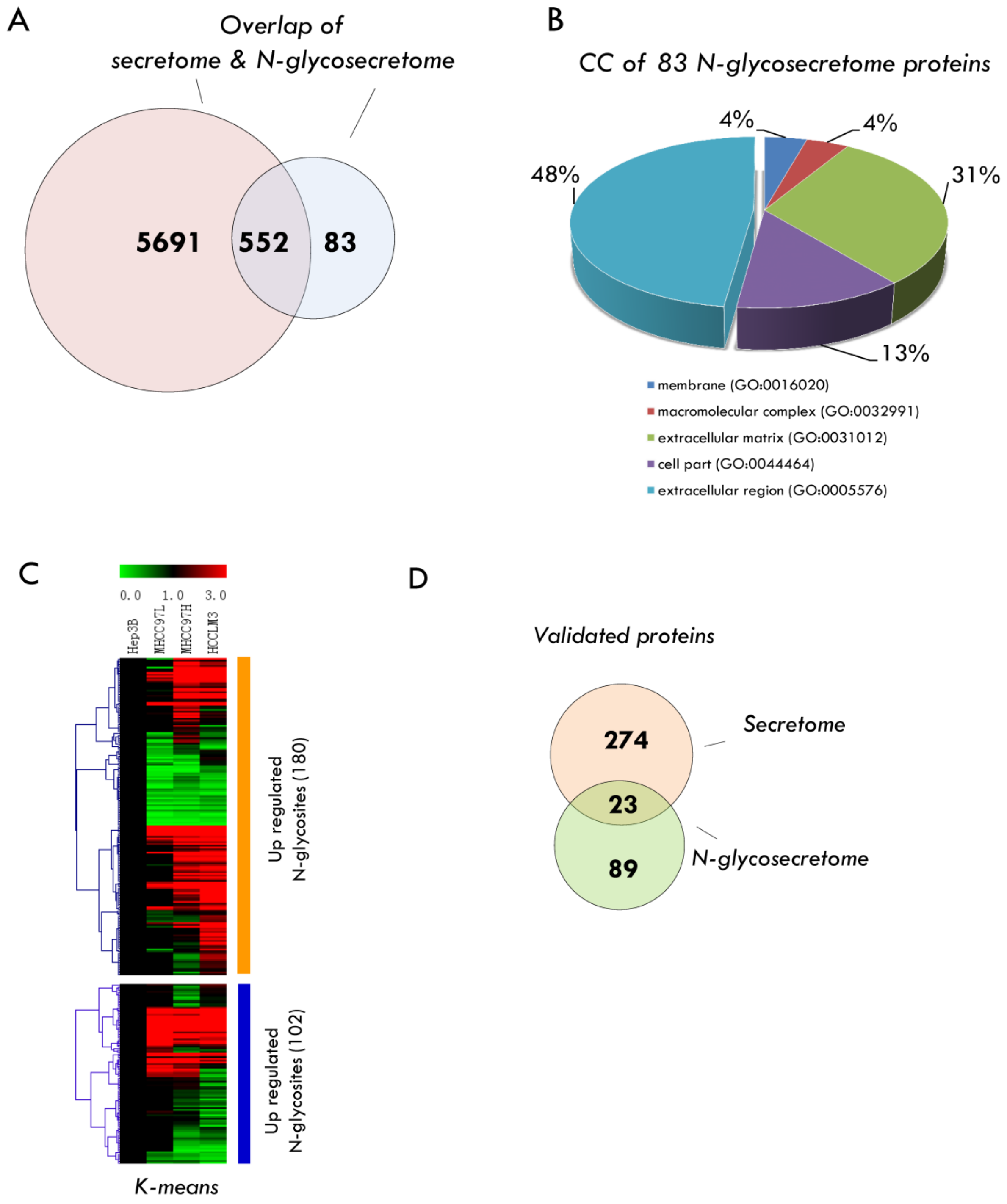

Supplemental Fig. S4

The Secretome versus the N-glycosecretome. A) The overlap of secretome and N-glycosecretome. B) Annotation of the new contribution from the N-glycosecretome. C) Hierarchical clustering and k-means analysis of the overlapped proteins from the secretome and the N-glycosecretome. D) The overlapped 23GPs were chosen as the validation candidates.

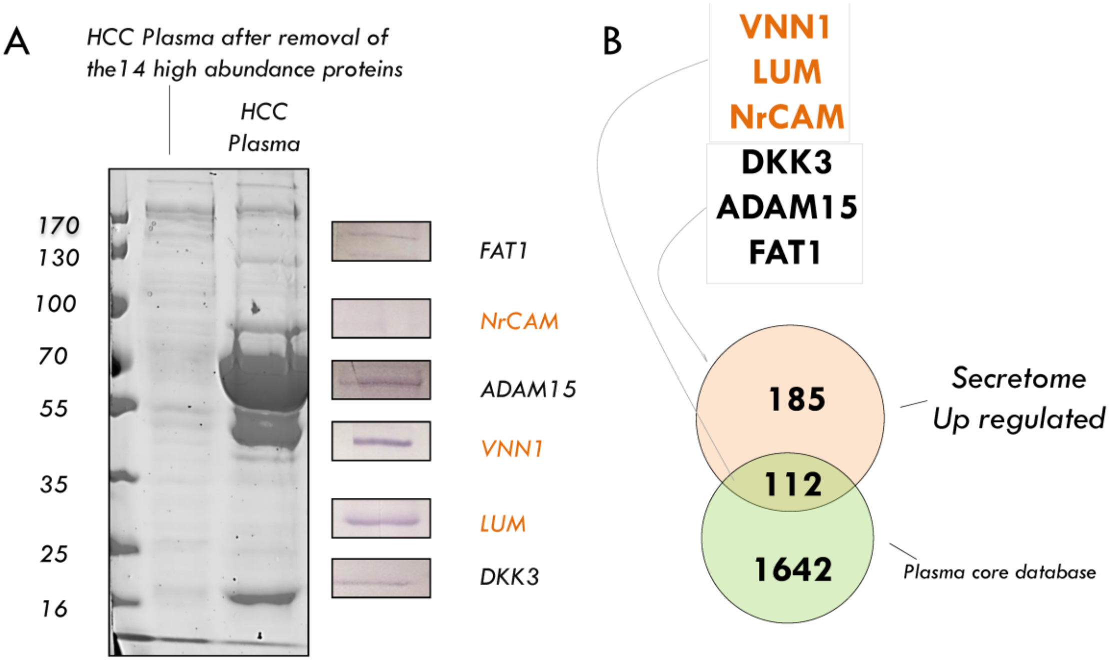

C

Protein Identifier	PSMs	Distinct Peptides	Estimated ng/mL	Coverage of Observable Sequence	Description	Gene Symbol
P51884	4069	44	4.0E+03	91%	Lumican	Lum
O95497	146	17	5.6E+01	53%	Pantetheinase	Vnn1
Q92823	44	12	1.6E+01	18%	Neuronal cell a	Nrcam

Supplemental Fig. S5

Validation of the candidates by western blotting in HCC plasma. A) The results of the candidates verified by western blotting in HCC plasma removed 14 high abundance proteins. B) A comparative analysis with the published core human plasma database (1754 GPs) revealed that there were 182 proteins not presented in the human plasma database but identified by our studies. C) The details of the three overlap proteins in core human plasma database and the secretome.
