# Supplementary material for: In-depth analysis of secretome and N-glycosecretome of human hepatocellular carcinoma metastatic cell lines shed light on metastasis correlated proteins
Source: Oncotarget. 2016 Mar 21;7(16):22031–49. doi: 10.18632/oncotarget.8247 (PMC5008342; doi:10.18632/oncotarget.8247)
Supplement: Supplementary file 8 [file oncotarget-07-22031-s008.pdf]

Supplemental Table 7: The relationship between the clinical pathological features of HCC and NrCAM.

Clinicopathological variables	Tumor NrCAM Expression		*P*-Value*
	Positive	Negative	
**Sex**			
Male	40	24	0.195
Female	4	7	
**Age**			
≤50	16	11	0.938
>50	28	20	
**Maximal tumor size**			
≤5cm	31	17	0.165
>5cm	13	14	
**Liver cirrhosis**			
Absent	26	19	0.848
Present	18	12	
**Venous invasion**			
Absent	37	30	0.354
Present	7	2	
**Tumor differentiation**			
Ⅰ - Ⅱ	7	2	
Ⅱ	16	27	0.002
Ⅱ-Ⅲ	3	20	
**pTNM Stage****			
Ⅰ	15	8	
Ⅱ	19	7	
ⅢA	7	4	0.030
ⅢB	3	5	
ⅢC-Ⅳ	0	7	

Supplemental Table 8: The relationship between the clinical pathological features of HCC and VNN1.

Clinicopathological variables	Tumor VNN1 Expression		*P*-Value*
	Positive	Negative	
**Sex**			
Male	35	29	0.093
Female	3	8	
**Age**			
≤50	12	15	0.419
>50	26	22	
**Maximal tumor size**			
≤5cm	29	19	0.024
>5cm	9	18	
**Liver cirrhosis**			
Absent	22	23	0.848
Present	16	14	
**Venous invasion**			
Absent	34	32	0.354
Present	4	5	
**Tumor differentiation**			
Ⅰ - Ⅱ	5	4	
Ⅱ	27	16	0.017
Ⅱ-Ⅲ	6	17	
**pTNM Stage****			
Ⅰ	15	8	
Ⅱ	14	12	
ⅢA	6	5	0.024
ⅢB	2	8	
ⅢC-Ⅳ	1	6	

Supplemental Table 9: The relationship between the clinical pathological features of HCC and LUM.

Clinicopathological variables	Tumor LUM Expression		*P*-Value*
	Positive	Negative	
**Sex**			
Male	35	29	0.571
Female	5	6	
**Age**			
≤50	15	12	0.772
>50	25	23	
**Maximal tumor size**			
≤5cm	28	20	0.247
>5cm	12	15	
**Liver cirrhosis**			
Absent	23	22	0.637
Present	17	13	
**Venous invasion**			
Absent	35	32	1.000
Present	5	4	
**Tumor differentiation**			
Ⅰ-Ⅱ	6	3	
Ⅱ	26	17	0.032
Ⅱ-Ⅲ	8	15	
**pTNM Stage****			
Ⅰ	15	8	
Ⅱ	14	12	
ⅢA	4	5	0.021
ⅢB	2	8	
ⅢC-Ⅳ	1	6	

Supplemental Table 10: The relationship between the clinical pathological features of HCC and ADAM15.

Clinicopathological variables	Tumor ADAM15 Expression		*P*-Value*
	Positive	Negative	
**Sex**			
Male	40	24	0.104
Female	4	7	
**Age**			
≤50	18	9	0.377
>50	27	21	
**Maximal tumor size**			
≤5cm	32	16	0.061
>5cm	12	15	
**Liver cirrhosis**			
Absent	27	18	0.848
Present	10	20	
**Venous invasion**			
Absent	37	29	0.379
Present	7	2	
**Tumor differentiation**			
Ⅰ-Ⅱ	3	6	
Ⅱ	29	14	0.126
Ⅱ-Ⅲ	12	11	
**pTNM Stage****			
Ⅰ	14	9	
Ⅱ	18	8	
ⅢA	6	5	0.016
ⅢB	6	2	
ⅢC-Ⅳ	0	7	

Supplemental Table 11: The relationship between the clinical pathological features of HCC and FAT1.

Clinicopathological variables	Tumor FAT1 Expression		*P*-Value*
	Positive	Negative	
**Sex**			
Male	34	30	0.750
Female	7	4	
**Age**			
≤50	14	13	0.713
>50	27	21	
**Maximal tumor size**			
≤5cm	29	19	0.182
>5cm	12	15	
**Liver cirrhosis**			
Absent	22	23	0.218
Present	19	11	
**Venous invasion**			
Absent	38	29	0.287
Present	3	6	
**Tumor differentiation**			
Ⅰ-Ⅱ	4	5	
Ⅱ	24	19	0.609
Ⅱ-Ⅲ	13	10	
**pTNM Stage****			
Ⅰ	16	7	
Ⅱ	21	5	
ⅢA	5	6	0.012
ⅢB	4	4	
ⅢC-Ⅳ	2	5	
